# Pharmacist-led remote follow-up service for non-metastatic breast cancer patients: a prospective randomised controlled trial of pharmaceutical intervention

**DOI:** 10.3389/fphar.2025.1640727

**Published:** 2025-09-04

**Authors:** Miaohui Wu, Xiaoyan Huang, Cuilv Liang, Peihong Wang, Yalan Zhang, Yin Zhang

**Affiliations:** ^1^ Department of Pharmacy, The Second Affiliated Hospital of Fujian Medical University, Quanzhou, China; ^2^ Department of Endocrinology, Quanzhou First Hospital Affiliated to Fujian Medical University, Quanzhou, China

**Keywords:** non-metastatic breast cancer, pharmacist, follow-up service, adverse drug reactions, quality of life

## Abstract

**Background:**

Non-metastatic breast cancer accounts for 87.8% of breast cancer cases. However, the high risk of drug adverse drug reactions due to multiple combined medications, along with the urgent need for out-of-hospital medication adherence and management, poses substantial challenges. Traditional treatments often fail to meet the full-cycle management needs of patients. Remote pharmaceutical follow-up, as an emerging model, may address these issues. This study aimed to investigate the intervention effect of remote pharmaceutical services via a follow-up app on non-metastatic breast cancer patients.

**Methods:**

From May 2023 to March 2025, 178 patients with non-metastatic breast cancer were enrolled and randomly assigned to an intervention group (receiving remote pharmaceutical follow-up via an app) or a control group (receiving only routine treatment), with a 6-month follow-up period. The primary outcome was the incidence of severe adverse drug reactions, while secondary outcomes included medication adherence scores, pharmaceutical knowledge scores, and quality of life assessments.

**Results:**

The incidence of severe adverse drug reactions in the intervention group (20.2%) was significantly lower than that in the control group (50.0%, *P* < 0.01), with notable improvements particularly in non-hematological adverse drug reactions. The intervention group also demonstrated significantly higher pharmaceutical knowledge scores and medication adherence scores compared to the control group (*P* < 0.05). In terms of quality of life, the intervention group showed greater improvements in symptom scores and overall composite scores (*P* < 0.01), with faster recovery of global health and functional scores in the late follow-up phase.

**Conclusion:**

Remote pharmaceutical services delivered via a follow-up app effectively reduce the incidence of severe adverse drug reactions and improve medication knowledge, adherence, and quality of life in non-metastatic breast cancer patients. This model provides a viable new approach for out-of-hospital management and highlights the value of pharmacists in the full-cycle management of oncology care. Future research should further explore preventive strategies for hematological adverse drug reactions and extend the follow-up period to refine the clinical application of this management model.

## 1 Introduction

According to data from the International Agency for Research on Cancer, in 2022, there were over 2.3 million new breast cancer cases globally, with approximately 670,000 deaths (Løyland et al., 2024). Non-metastatic breast cancer accounts for 87.8% of all breast cancer cases, comprising the majority of the breast cancer population (Morgan et al., 2024). However, the American Cancer Society has noted significant gaps and deficiencies in post-treatment follow-up and monitoring for breast cancer patients, with most cancer patients unable to receive optimal diagnostic and treatment guidance (Siegel et al., 2023). This situation exacerbates the complexity of breast cancer management, particularly given the large population of non-metastatic breast cancer patients who require long-term intervention, urgently necessitating more effective solutions.

At the treatment level, breast cancer patients often receive multiple combined medications, significantly increasing the risk of potential adverse drug reactions. Common adverse drug reactions such as nausea and vomiting not only severely affect patients’ medication adherence and quality of life but may even lead to treatment failure (Roscoe et al., 2012; Lau et al., 2016; Wang et al., 2023). Studies have shown that over 70% of patients experience symptoms such as nausea and diarrhea after receiving various treatment regimens, and these may trigger psychological sub-health issues like anxiety and fatigue, further weakening treatment efficacy (Hashimoto et al., 2020; Yeo et al., 2020). Additionally, after discharge, patients often face problems such as non-adherence to medications, incorrect dosing, and drug incompatibilities, especially when taking anticancer drugs like capecitabine, pyrotinib, and cyclophosphamide. Patients have significantly less convenient access to monitoring and treatment services when outside the hospital compared to during hospitalization (Sweetman et al., 2014). Although the 5-year survival rate for non-metastatic breast cancer patients has increased to 83.1% (Ho et al., 2018), this has also raised higher requirements for drug therapy and health management. Patients face continuously increasing medication-related problems and treatment needs, requiring interventions that span long-term or even lifelong periods (Papakonstantinou et al., 2025). These challenges highlight the necessity of systematic medication management and patient support.

Pharmaceutical care, since its proposal by American pharmacotherapy scholars Hepler and Strand, has undergone 30 years of development, with its core focus on providing responsible drug-related services to the public with the goal of improving quality of life (Hepler and Strand, 1990). During this period, the role of pharmacists has gradually transformed, meeting patients’ full-cycle diagnostic and treatment needs by providing professional medication guidance, coordinating physician-patient communication, and formulating personalized medication recommendations.

Pharmacist-led patient education programs, as a core component of this transformation, have gained widespread recognition for their ability to enhance medication safety and adherence. These educational initiatives operate primarily through two distinct models, each with its own characteristics. The traditional in-person education model enables direct interaction: pharmacists can visually assess patients’ conditions, engage in face-to-face communication, and address treatment-related issues in real time (Ma et al., 2025). However, this approach is constrained by geographical barriers, fixed schedules, and resource intensity, which prevent homebound patients or those in remote areas from accessing timely guidance.

In contrast, remote education programs leverage digital platforms to overcome these limitations. They offer flexibility in terms of timing and accessibility, allowing patients to access educational materials, consult pharmacists via text or video, and receive continuous follow-up (Gushrowski et al., 2025; Metrebian et al., 2025)—an advantage for the long-term management of cancer. Nevertheless, remote models present their own challenges: the lack of in-person interaction may diminish trust to some extent, and technical barriers (such as software usage) could potentially exclude vulnerable populations like elderly patients (Kanimozhi et al., 2025).

While face-to-face services excel in personalized care, remote models provide broader reach and sustained engagement, making them particularly suitable for the out-of-hospital management of non-metastatic breast cancer—a population requiring long-term, convenient service support. In recent years, pharmacist-led remote service models have rapidly developed and gained widespread recognition globally (McNab et al., 2018; Abousheishaa et al., 2020; Gonçalves et al., 2021; Ruiz-Ramos et al., 2021; Kc et al., 2023), offering new pathways to address the management challenges of breast cancer patients.

Against this background, this study conducted a prospective, randomized controlled clinical trial for non-metastatic breast cancer patients, aiming to explore a new model of remote pharmaceutical follow-up services.

## 2 Materials and methods

This study was conducted in the Department of Breast Surgery, The Second Affiliated Hospital of Fujian Medical University, from May 2023 to March 2025. All participants provided written informed consent after being fully informed of the study objectives, methods, potential risks, and benefits. The study was approved by the hospital ethics committee (approval number: [2023] Ethic Review No. (288)) and registered with the Chinese Clinical Trial Registry (registration number: ChiCTR2300078817). The study design and methods adhered to the principles of the Declaration of Helsinki and its 1964 revision.

### 2.1 Inclusion criteria

Patients were included if they met the following criteria: (1) newly diagnosed with non-metastatic breast cancer (AJCC stages 0-III); (2) aged 18–80 years; (3) expected survival >6 months; (4) capable of communication, without significant cognitive impairment, and able to use a smartphone; (5) willing to cooperate with long-term follow-up and attend scheduled follow-up visits.

### 2.2 Exclusion criteria

Patients were excluded if they had: (1) a history of other primary malignancies; (2) received prior treatment (surgery, radiotherapy, chemotherapy, etc.) at external hospitals; (3) difficulties in verbal communication; (4) participation in other concurrent clinical trials.

### 2.3 Study design

**FIGURE 1:**
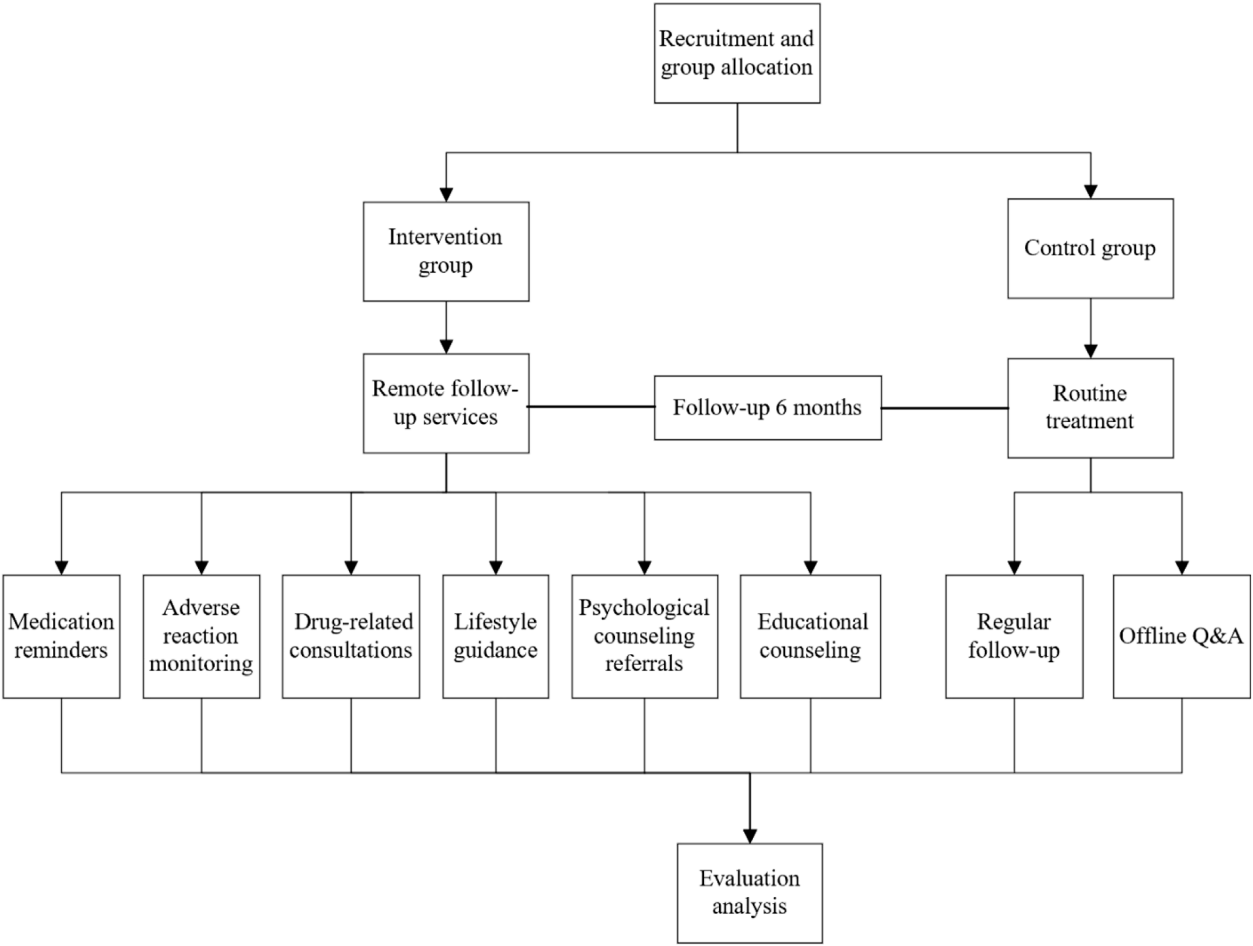
Study design framework.

This was a prospective, randomized, single-blind pilot clinical study designed to evaluate the safety and efficacy of pharmacist-provided remote follow-up services in patients with non-metastatic breast cancer. The study utilized a 1:1 randomized controlled design, with participants randomly assigned to either the intervention group or the control group, and followed up for 6 months. Participants were enrolled after postoperative pathological reports confirmed their eligibility (meeting the inclusion criteria). Upon the eligible patients agreeing to participate, the recruiting pharmacist requested a random number from an independent pharmacist who maintained the random number table (pre-registered and filed with the hospital’s Clinical Research Office prior to trial initiation). A random number of 1 indicated assignment to the intervention group, and 2 indicated assignment to the control group. The 6-month follow-up period started immediately after the signing of the informed consent form. During hospitalization, both groups received traditional pharmaceutical services (including personal file establishment and medication education). After discharge, the intervention group received follow-up services from pharmacists via remote software (such as adverse reaction monitoring, online consultations, and review reminders), with interventions for non-hematological toxicities structured according to the Chinese Breast Cancer Follow-up and Health Management Guideline, which provides evidence-based recommendations for symptom management (DOI: 10.3760/cma.i.cn112152-20211029-00798), while the control group did not receive follow-up services (See Figure 1 for the study design framework).

As per standard oncology practice, the duration of hospitalization and frequency of outpatient visits for both groups were determined by clinical needs (e.g., treatment-related complications, disease progression). Pharmacists did not intervene in clinical decisions related to these parameters; instead, their role was centered on providing professional recommendations (e.g., medication-related advice), thereby ensuring the independence and standardization of clinical care throughout the study.

### 2.4 Study software and intervention measures

The follow-up software used in this study is “Haixin Health,” an intelligent application developed by Zhejiang Haixin Zhihui Technology Company Limited, which is designed specifically for oncology patients with multi-dimensional functionalities. Patients have full access to their medical records via the software at any time and from anywhere, and upon uploading these records, the system generates preliminary diagnostic and treatment recommendations through AI analysis. These recommendations are then double-checked by physicians and pharmacists to formulate personalized treatment plans. It dynamically organizes patients’ treatment histories in a timeline format, integrating key information such as examination reports and medication records to help patients quickly grasp their condition. The software supports multimodal interactions (e.g., text, telephone, video) to enable remote consultations and communication between patients, research pharmacists, and oncology experts. Additionally, it establishes an exclusive communication community where patients can share treatment experiences, dietary restrictions, and daily life insights to facilitate mutual support and rehabilitation management. As visualized in Supplementary File 1 (screenshots of the software interface attached), the community module integrates seamlessly with patient consultation records and condition-tracking features. All enrolled patients were confirmed to have the ability and conditions to use the research software. Upon enrollment, they received dedicated guidance on software operation, which included navigating community interactions, using consultation functions, and accessing medical records, to ensure full mastery of all features.

Research pharmacists completed a 3-month standardized intervention training program at the Pharmacy Department and passed competency assessments prior to participating in the trial, providing each patient with 6 months of structured follow-up services. During the follow-up period, pharmacists regularly updated and evaluated medication lists based on patients’ treatment progress, and used online questionnaires to assess medication adherence and treatment-related knowledge. Guided by these assessment results, they conducted targeted follow-ups at least once every 2 weeks. Leveraging the “Haixin Health” platform, pharmacists could set up automated reminders, which the software sent to patients at scheduled intervals to prompt medication intake and follow-up appointments. Continuous monitoring of adverse reactions was also implemented via the platform: whenever patients experienced treatment-related adverse events of any grade, they could report symptoms to pharmacists, and receive preventive guidance and intervention recommendations after consultation. Beyond adverse reaction monitoring, real-time services encompassed medication consultations, lifestyle guidance (exercise/nutrition plans), psychological counseling referrals, and shared educational resources (such as dietary arrangements during chemotherapy, skin care during radiotherapy, and postoperative wound care) to support rehabilitation.

Evaluations were conducted at baseline, 3 months, and 6 months to dynamically adjust intervention strategies. Such personalized guidance and ongoing interactive engagement helped maintain close connections with patients to the greatest extent.

### 2.5 Sample size estimation

Referencing relevant literature (Li et al., 2021), we set the incidence of severe adverse drug reactions in the control group as $P_{A}$ = 50% and in the intervention group as $P_{B}$ = 30%, expecting to observe an improvement in incidence of $P_{A}-P_{B}=$ 20%. With a one-tailed test level of α = 5%, $Z_{1-\alpha }$ = 1.645 and statistical power 1−β = 80%, $Z_{1-\beta }$ = 0.84, the formula for estimating the sample size based on the comparison of two sample rates (using severe adverse reaction incidence as the outcome indicator) is as follows:

$$n_{A}={\left(\frac{Z_{1-\alpha }+Z_{1-\beta }}{P_{A}-P_{B}}\right)}^{2}\times \left(P_{A}\left(1-P_{A}\right)+P_{B}\left(1-P_{B}\right)\right)$$

The calculated sample size was $n=n_{A}+n_{B}=71\times 2=142$ . Considering a 20% dropout rate, the estimated sample size was adjusted to 142/0.8 ≈ 178.

### 2.6 Endpoints and data acquisition

#### 2.6.1 Primary endpoint

The primary outcome was the incidence of adverse drug reactions, defined in accordance with the National Cancer Institute Common Terminology Criteria for Adverse Events (NCI-CTCAE) v5.0. This standardized framework grades adverse events on a 1–5 scale: grade 1 (mild, requiring no intervention), grade 2 (moderate, necessitating local intervention), grade 3 (severe, warranting hospitalization), grade 4 (life-threatening), and grade 5 (fatal). Severe adverse reactions were specifically categorized as grade 3 or higher, consistent with clinical oncology standards. A dichotomous approach was used to assess the correlation between adverse reactions and treatment, with only “related” cases included in the analysis.

#### 2.6.2 Secondary endpoints

1. Medication adherence scores, evaluated using the MMAS-8 scale. This scale assesses dimensions such as medication behavior, attitudes, habits, and difficulties, with a scoring range of 0–8 points (higher scores indicate better adherence) (Muntner et al., 2011; Kwan et al., 2020).
2. Breast cancer medication knowledge scores, based on a questionnaire independently designed by the research team. We first referred to the Breast Cancer Awareness Measure (Breast CAM) questionnaire (Linsell et al., 2010), which focuses on core disease - related knowledge. Then, we integrated common clinical issues encountered by breast cancer patients in practice.

This customized tool covers key aspects such as breast cancer etiology, symptoms, diagnosis, medication treatment, and adverse reaction prevention (consistent with the foundational framework of Breast CAM). It also includes specific items from our “Breast Cancer Knowledge Quiz” (see Supplementary File 3 for the full questionnaire), like whether to avoid estrogen - containing drugs during treatment, the impact of controlling high - risk factors on breast cancer risk, and precautions for medication use (e.g., self - use of medicine, timing of chemotherapy - related treatments). The scoring range is 0–20 points, with higher scores indicating a better grasp of both theoretical knowledge and practical medication skills relevant to breast cancer patients.

3. Quality of life scores, assessed using the European Organization for Research and Treatment of Cancer (EORTC) QLQ-C30 scale, which evaluates four dimensions: global health, functional scores, symptom scores, and overall composite scores.

### 2.7 Data acquisition

Data collection for the endpoints was conducted as follows: The electronic questionnaires related to the trial were first distributed to patients. For the intervention group, these questionnaires were distributed online via the “Haixin Health” software, while the control group received them through email/SMS. Patients then completed the data entries into the electronic questionnaires. Additionally, paper-based questionnaires were provided to accommodate patients who preferred or had difficulty completing electronic versions, ensuring offline accessibility. All collected data were uniformly stored in the software’s supporting management platform (Supplementary File 2). The data stored in the software platform was managed and maintained by independent pharmacists from a third party, with the generated data being non-editable and non-modifiable to ensure data integrity and reliability.

### 2.8 Statistical analysis

All case data in this study were analyzed using IBM SPSS Statistics 27.0 software. Measurement data with normal distribution were expressed as mean ± standard deviation ( $\bar{x}\pm s$ ), while non-normally distributed data were presented as median (quartile). For small-sample count data (*n* ≤ 20), frequencies were used for representation; general count data (*n* > 20) were expressed as percentages. The chi-square test ( $\chi^{2}$ ) was used to compare the incidence of adverse drug reactions between the two groups. Indicators such as quality-of-life scores, medication knowledge scores, and medication adherence scores were analyzed using two-way repeated measures analysis of variance (Two-way Repeated Measures ANOVA) to evaluate the intervention effect by assessing the interaction between intervention factors and time. In all statistical tests, a significance level of *P* < 0.05 was adopted, with a two-sided 95% confidence interval. To minimize potential bias, the study was supervised by the hospital ethics committee throughout the process and underwent regular data verification and quality control by the Clinical Research Office. Data analysis was conducted by third-party statisticians.

## 3 Results

### 3.1 Basic study information

**FIGURE 2:**
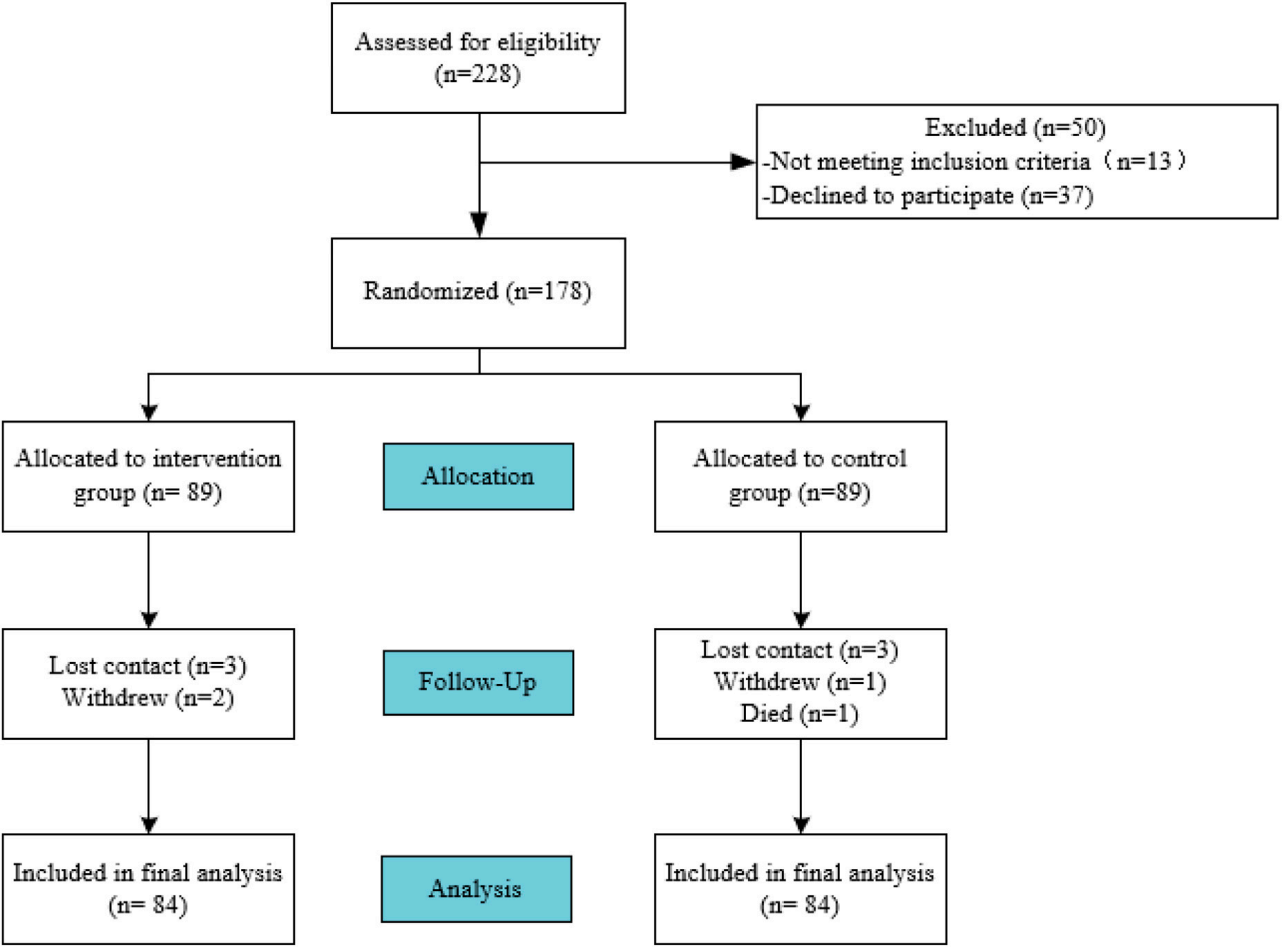
Flowchart of study participant enrollment.

During the study period, a total of 228 patients were screened, among whom 13 met the exclusion criteria, 37 refused to participate, and 178 patients signed informed consent to be included in the study, with 89 patients in each group. During the follow-up period, 10 subjects were lost to follow-up, and finally 168 patients completed the six-month follow-up study. The flowchart of participant enrollment is shown in Figure 2.

After obtaining informed consent, Pharmacists immediately created and maintained detailed patient files upon obtaining informed consent, with continuous updates of patient information throughout the follow-up period. Basic information such as age, BMI, surgical procedures, and treatment regimens, hospitalization duration, outpatient visits frequency were collected according to the trial protocol, as detailed in Table 1.

**TABLE 1:** Table of patients’ baseline characteristics.

Characteristics	Intervention (n = 84)	Control (n = 84)	*P* value
Age, years	45.9 ± 8.1	47.3 ± 8.9	0.306
BMI	22.8 ± 3.3	23.5 ± 3.2	0.116
Cancer Stages			0.248
0	10	5	
1	30	25	
2	37	41	
3	7	13	
Molecular Subtype			0.995
Luminal A	22	21	
Luminal B	43	43	
HER2+	9	10	
Triple-negative	10	10	
Surgical Approaches			0.915
Lumpectomy	3	2	
Breast-conserving modified radical mastectomy	33	30	
Modified radical mastectomy	4	4	
Radical resection	44	48	
Surgical Site			0.107
Left	48	39	
Right	34	45	
Bilateral	2	0	
Lymph node dissection	22	30	0.182
Treatment Regimen^a^			
Chemotherapy	57	57	1.000
Radiotherapy	35	37	0.755
Endocrine therapy	35	33	0.753
Targeted therapy	15	11	0.394
Drug Category			0.755
Self-funded drugs	5	6	
Hospital-provided drugs	79	78	
Chronic Diseases			
Diabetes mellitus	3	2	0.650
Hypertension	3	7	0.192
Hepatitis B	7	5	0.549
Hyperthyroidism	1	2	0.560
Asthma	1	1	1.000
Systemic lupus erythematosus	0	1	0.316
Fatty liver disease	2	0	0.155
Multimorbidity (≥2 chronic diseases)	1	3	0.311
Medical insurance			0.378
Employee Medical Insurance	14	10	
Resident Medical Insurance	70	74	
Educational Attainment			0.320
Primary education	41	48	
Secondary education	33	31	
Tertiary education	10	5	
Menopause	26	32	0.330
Employment	74	66	0.098
Married	82	83	0.560
Hospitalization Duration, days	5.6	5.8	0.340
Outpatient Visits Frequency, times	8.7	9.1	0.221

^a^ Treatment regimens reflect actual therapies received during the study.

### 3.2 Incidence of adverse drug reactions

During the 6-month clinical trial, we compared the incidence of adverse drug reactions between the two groups in different categories, as shown in Table 2.

**TABLE 2:** Comparison of serious adverse drug reactions between two groups of patients.

Adverse reaction category	Intervention (n = 84)	Control (n = 84)	Total (n = 168)	*P* value
Patients with serious adverse drug reactions	17 (20.2%)	42 (50.0%)	59 (35.1%)	<0.001
Hematological only	8 (9.5%)	8 (9.5%)	16 (9.5%)	1.000
Non-hematological only	7 (8.3%)	21 (25.0%)	28 (16.7%)	0.004
Both hematological and non-hematological	2 (2.4%)	13 (15.5%)	15 (8.9%)	0.007
Types of serious adverse drug reactions (with incidence in ≥2% of patients)*				
Hematological				
Leukopenia	10 (11.9%)	18 (21.4%)	28 (16.7%)	NA
Neutropenia	1 (1.2%)	4 (4.8%)	5 (3.0%)	NA
Non-hematological				
Nausea	3 (3.6%)	14 (16.7%)	17 (10.1%)	NA
Insomnia	2 (2.4%)	10 (11.9%)	12 (7.1%)	NA
Wound infection	1 (1.2%)	5 (6.0%)	6 (3.6%)	NA

In the intervention group, 17 out of 84 patients (20.2%) experienced severe adverse drug reactions, compared with 42 out of 84 patients (50.0%) in the control group. The intervention group had a significantly lower incidence of severe adverse drug reactions than the control group (*P* < 0.001). No significant difference was observed between the two groups in the incidence of hematological adverse drug reactions alone (*P =* 1.000). The intervention group showed lower incidences in non-hematological adverse drug reactions alone, as well as both hematological and non-hematological adverse drug reactions, with statistically significant differences between the two groups (*P =* 0.004 and *P =* 0.007, respectively). The most common severe hematological adverse reaction was leukopenia (overall incidence 16.7%), and the most common severe non-hematological adverse reaction was nausea (overall incidence 10.1%).

Additionally, we recorded the occurrence of adverse drug reactions (all grades, 1–5) during the trial. The adverse reaction profiles of the two groups are detailed in Table 3.

**TABLE 3:** Total record of adverse drug reactions in the two groups.

Adverse reaction category		Intervention (n = 84)	Control (n = 84)
Hematological			
	Leukopenia	35	39
	Anemia	15	22
	Neutropenia	9	11
Total cases		59	72
Non-hematological			
	Nausea	21	42
	Alopecia	21	33
	Hypercholesterolemia	24	24
	Hyperuricemia	13	25
	Vomiting	13	23
	Insomnia	15	20
	Hypertriglyceridemia	15	12
	Wound pain	10	17
	Rash	11	14
	Wound infection	6	17
	Drug-induced liver injury	4	11
	Hyperglycemia	5	11
	Hematuria	1	5
	Fever	3	1
	Constipation	1	2
Total cases		163	257

Patients may experience more than one type of adverse reaction, so the total number of cases does not equal the total number of patients.

### 3.3 Medication knowledge

**FIGURE 3:**
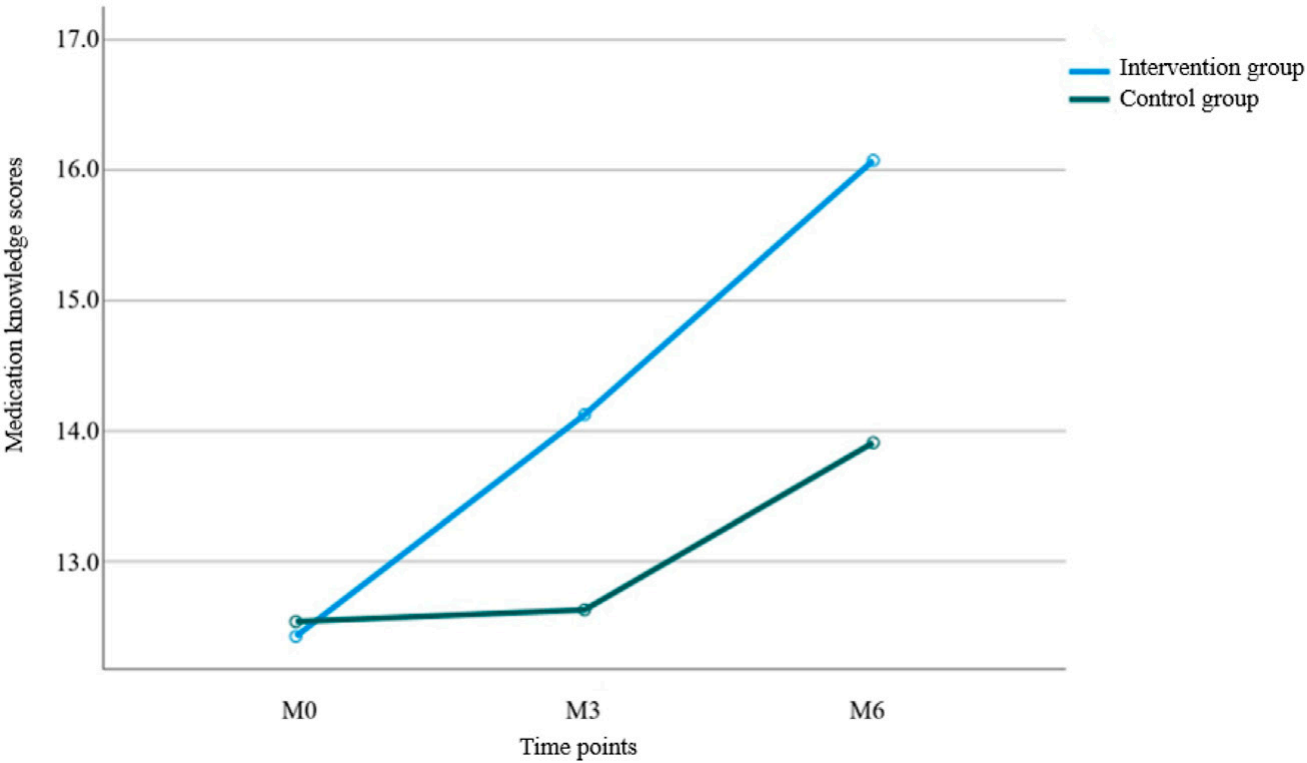
Trend of the scores in medication knowledge between the two groups.

Results from Table 4 and Figure 3 indicated statistically significant differences in medication knowledge scores between the two groups (*P* < 0.001 for intergroup comparison). Significant differences were also observed in knowledge scores across different time points (*P* < 0.001 for intragroup comparison), with a significant interaction effect between group and time (*P* < 0.001 for group × time), suggesting that medication knowledge scores varied across groups, time points, and their rates of change. Pairwise comparisons showed: M0<M3<M6 over time, indicating a continuous increase in knowledge scores from baseline to 6 months of follow-up, reaching the highest value at 6 months. The intervention group demonstrated a faster increase in scores from M0 to M3 compared to the control group, while no significant difference was observed between the two groups from M3 to M6.

**TABLE 4:** Repeated measures ANOVA results of the medication knowledge scores of the two groups.

Source of variation	Sum of squares	Degrees of freedom	Mean square	F value	*P* value
Between groups (Group)	55.242	1	55.242	13.702	<0.001
Within groups (Time))	511.677	1.875	272.945	156.801	<0.001
Group × Time interaction	108.335	1.875	57.790	33.199	<0.001
Subjects (nested within Group)	1886.763	156	12.095		
Error	509.062	292.445	1.741		
Total	3,071.079	453.195			

### 3.4 Medication adherence

**FIGURE 4:**
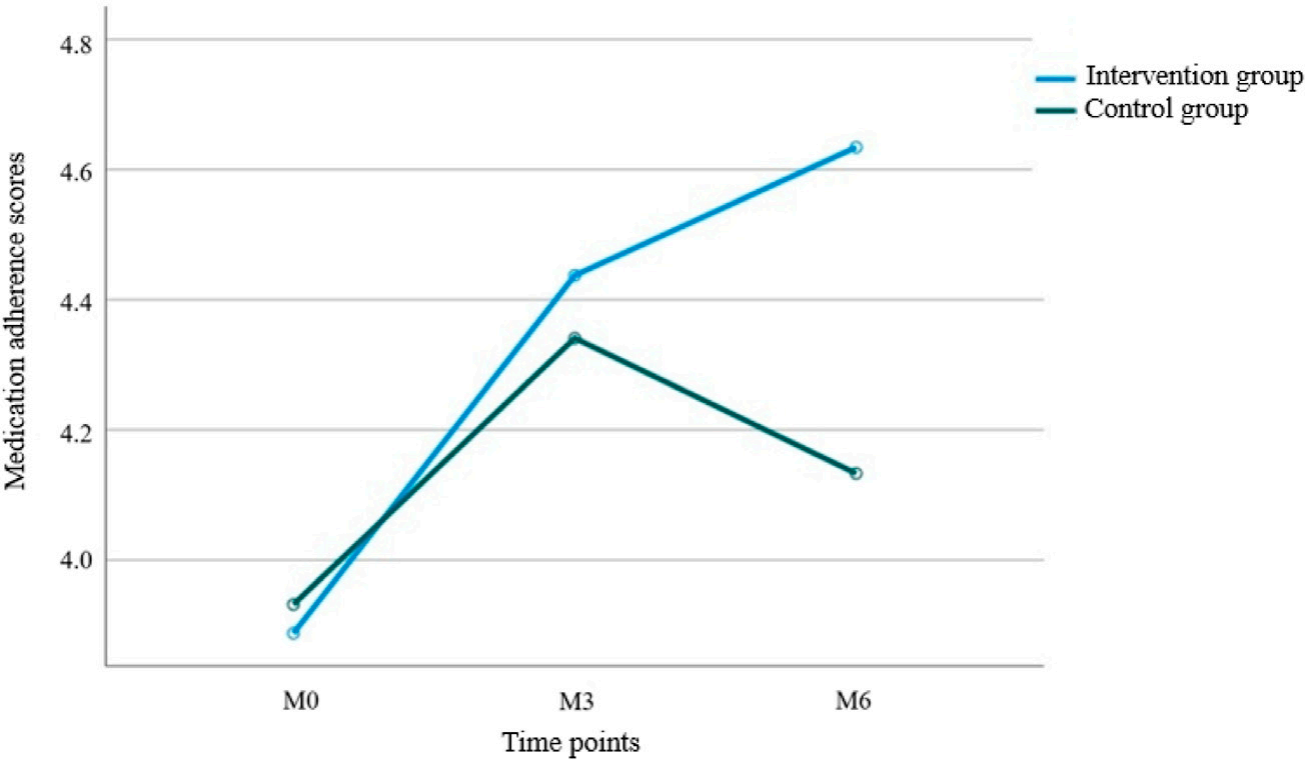
Trend of medication adherence scores between the two groups.

Results from Table 5 and Figure 4 showed statistically significant differences in medication adherence scores between the two groups (*P =* 0.047 for intergroup comparison). Significant differences were also observed in adherence scores across different time points (*P =* 0.002 for intragroup comparison), with a significant interaction effect between group and time (*P =* 0.021 for group × time), indicating that medication adherence scores varied across groups, time points, and their rates of change. Pairwise comparisons revealed: M0<M3 = M6 over time, suggesting a continuous increase in adherence scores from baseline to M3, followed by stabilization. The intervention group exhibited faster rates of change in adherence scores during both the M0–M3 and M3–M6 periods compared to the control group, with statistically significant differences.

**TABLE 5:** Repeated measures ANOVA results of adherence scores between two groups.

Source of variation	Sum of squares	Degrees of freedom	Mean square	F value	*P* value
Between groups (Group)	3.250	1	3.250	4.012	0.047
Within groups (Time)	24.801	2	12.401	6.592	0.002
Group × Time interaction	14.809	2	7.405	3.936	0.021
Subjects (nested within Group)	270.346	155	1.744		
Error	583.167	310	1.881		
Total	896.373	470			

### 3.5 Quality of life

**FIGURE 5:**
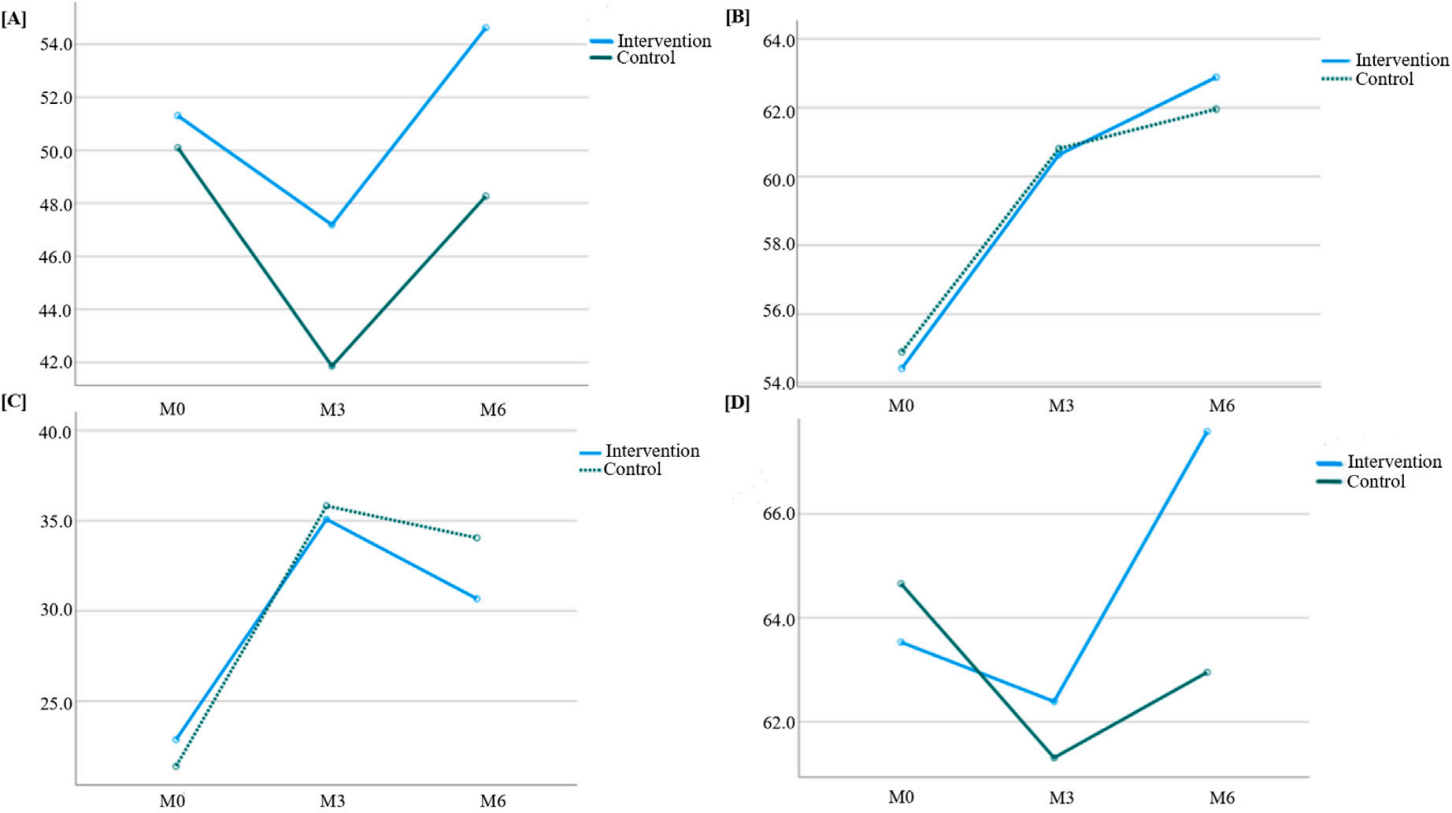
Trend of quality-of-life scores over time between the two groups. **(A)** Global health; **(B)** Functional score; **(C)** Symptom score; **(D)** Overall score.

Four dimensions of quality of life were evaluated: global health, functional scores, symptom scores, and overall composite scores. Each score was converted to a 100-point scale, where higher symptom scores indicated more symptoms and poorer quality of life, while higher scores in the other three dimensions reflected better quality of life. The scoring statistics are shown in Table 6, and the trends of quality-of-life scores over time in both groups are illustrated in Figure 5.

**TABLE 6:** Statistics of quality of life scores at different times in the two groups.

Dimension	Groups	Number of cases	M0	M3	M6
Global health	Intervention	83	51.3 ± 16.2	43.3 ± 19.7	54.6 ± 15.5
	Control	81	50.1 ± 16.5	41.4 ± 15.8	48.3 ± 12.3
Functional scores	Intervention	83	54.4 ± 7.2	60.6 ± 7.4	62.9 ± 7.2
	Control	81	54.9 ± 6.9	60.8 ± 6.2	61.9 ± 6.1
Symptom scores	Intervention	83	22.8 ± 6.5	35.1 ± 9.9	30.7 ± 8.3
	Control	81	21.4 ± 8.0	35.8 ± 11.3	34.1 ± 10.6
Overall composite scores	Intervention	83	63.5 ± 6.0	62.4 ± 6.0	67.6 ± 5.1
	Control	81	64.7 ± 5.9	61.3 ± 5.5	63.0 ± 4.9

Results from Table 7 indicated statistically significant differences in global health scores between the two groups (*P =* 0.028 for intergroup comparison). Significant differences were also observed in global health scores across different time points (*P* < 0.001 for intragroup comparison), though no significant interaction effect was found between group and time (*P =* 0.115 for group × time), suggesting that global health scores varied across groups and time points. Pairwise comparisons showed that global health scores decreased continuously from M0 to M3 in both groups, reaching the lowest value at M3, then increased from M3 to M6 and returned to baseline levels.

**TABLE 7:** Results of repeated measures ANOVA for global health scores in the two groups.

Source of variation	Sum of squares	Degrees of freedom	Mean square	F value	*P* value
Between groups (Group)	761.635	1	761.635	4.917	0.028
Within groups (Time)	4,755.129	2	2,377.565	16.935	<0.001
Group × Time interaction	611.864	2	305.932	2.179	0.115
Subjects (nested within Group)	24,660.408	163	151.291		
Error	45,769.456	326	140.397		
Total	76,558.492	494			

Table 8 showed no statistically significant difference in functional scores between the two groups (*P =* 0.788 for intergroup comparison), but significant differences were observed across time points (*P* < 0.001 for intragroup comparison), with no interaction effect between group and time (*P =* 0.293 for group × time). This indicated that functional scores varied across time points but not between groups. Pairwise comparisons revealed a trend of M0<M3<M6 for functional scores in both groups, meaning scores gradually increased over time and reached the highest value at M6.

**TABLE 8:** Results of repeated measures ANOVA for functional scores in the two groups.

Source of variation	Sum of squares	Degrees of freedom	Mean square	F value	*P* value
Between groups (Group)	2.718	1	2.718	0.073	0.788
Within groups (Time)	5,508.959	1.738	3,169.101	180.045	<0.001
Group × Time interaction	37.320	1.738	21.469	1.220	0.293
Subjects (nested within Group)	18,285.647	163	112.182		
Error	4,987.432	283.349	17.602		
Total	28,822.076	450.825			

According to Table 9, no significant intergroup difference was found in symptom scores (*P =* 0.454), but significant differences were observed across time points (*P* < 0.001), with a significant interaction effect between group and time (*P* = 0.005). This suggested that symptom scores varied across time points and showed different change rates between groups. Pairwise comparisons showed M3>M6>M0 for symptom scores, indicating an upward trend from M0 to M3, followed by a decline from M3 to M6 (though still higher than baseline). The intervention group exhibited a slower increase in symptom scores from M0 to M3 and a faster decrease from M3 to M6 compared to the control group, with statistically significant differences.

**TABLE 9:** Results of repeated measures ANOVA for symptom scores in the two groups.

Source of variation	Sum of squares	Degrees of freedom	Mean square	F value	*P* value
Between groups (Group)	32.191	1	32.191	0.564	0.454
Within groups (Time)	16,252.322	1.551	10,477.677	187.437	<0.001
Group × Time interaction	541.904	1.551	349.359	6.250	0.005
Subjects (nested within Group)	27,919.848	163	171.287		
Error	14,133.411	252.835	55.900		
Total	58,879.676	419.937			

Table 10 showed statistically significant differences in overall composite scores between the two groups (*P =* 0.038 for intergroup comparison), with significant variations across time points (*P* < 0.001 for intragroup comparison) and a significant interaction effect between group and time (*P* < 0.001 for group × time). This indicated that overall composite scores differed across groups, time points, and change rates. Pairwise comparisons revealed M3<M0<M6 for overall composite scores, meaning scores decreased rapidly from M0 to M3 and then increased continuously from M3 to M6. The intervention group showed a slower decline in overall composite scores from M0 to M3 and a faster increase from M3 to M6 compared to the control group, with statistically significant differences.

**TABLE 10:** Results of repeated measures ANOVA for the Overall composite scores in the two groups.

Source of variation	Sum of squares	Degrees of freedom	Mean square	F value	*P* value
Between groups (Group)	96.618	1	96.618	4.367	0.038
Within groups (Time)	996.673	2	498.336	37.164	<0.001
Group × Time interaction	696.504	2	348.252	25.971	<0.001
Subjects (nested within Group)	10,818.986	163	66.374		
Error	4,371.364	326	13.409		
Total	16,980.145	494			

## 4 Discussion

In the evaluation of oncology treatment efficacy, multiple indicators need to be considered. Compared with evaluation metrics such as objective response rate, disease control rate, and overall survival in cancer patients, adverse drug reactions are more easily captured and directly benefit breast cancer patients (Dowling et al., 2023). Therefore, the incidence of adverse drug reactions was set as the primary outcome indicator in this study. Previous studies have shown that the incidence of severe treatment-related adverse drug reactions in breast cancer is approximately 18%–66% (Poggio et al., 2018; Shen et al., 2025). The results of this study showed that the incidence of severe adverse drug reactions in the intervention group was 20.2%, significantly lower than 50.0% in the control group (*P* < 0.001), indicating that the intervention measures were significantly effective in reducing the risk of adverse drug reactions.

No significant difference was observed between the two groups in hematological adverse drug reactions alone, suggesting that the intervention may primarily act through non-hematological pathways, which is consistent with the findings of Li et al. (2021). The intervention group had significantly lower incidences of non-hematological adverse drug reactions alone and combined hematological and non-hematological adverse drug reactions compared with the control group (*P =* 0.004 and *P =* 0.007, respectively). These results indicate the potential advantages of the intervention in reducing non-hematological adverse drug reactions and suggest that the significant differences in overall outcomes may be attributed to improvements in non-hematological adverse drug reactions. The most common severe hematological adverse reaction in this study was leukopenia, with an incidence of 16.7%. However, in clinical practice, due to restrictions of medical policies, researchers typically only intervene when a patient’s white blood cell count is lower than 4 × 10^9^/L. Although this practice is consistent with clinical routines, its preventive effect is relatively limited. As Huan Wang et al. pointed out in their study (Wang et al., 2025), myelosuppression, as a dose-limiting toxicity, often requires improvement through drug dose adjustment or recombinant human growth factor support. Therefore, interventions for hematological adverse drug reactions often have a certain degree of lag.

In contrast, non-hematological adverse drug reactions offer more room for intervention. In this study, patients in the intervention group received more systematic and personalized pharmaceutical follow-up services. Pharmacists regularly communicated with patients to inquire in detail about their medication use and physical condition, promptly identifying and addressing potential adverse drug reactions. This model is highly consistent with the medication therapy management services recommended by the American Pharmacists Association (Guo et al., 2024). For example, when patients experienced adverse drug reactions such as nausea and vomiting, those in the intervention group generally followed pharmacists’ advice to use symptom-improving medications early, regularly, and in sufficient doses. Multiple guidelines have indicated that such proactive monitoring and intervention help reduce the severity of adverse drug reactions, prevent symptom progression, and thereby lower the incidence of severe adverse drug reactions (McKenzie et al., 2019; Aapro et al., 2021; Kennedy et al., 2024).

Second, intervention group patients received more medication guidance and health education during follow-up, enhancing their awareness of drug adverse drug reactions and enabling them to take timely measures when reactions occurred. For instance, when hair loss occurred, patients wore ice caps prior to subsequent chemotherapy, which helped reduce the incidence of severe hair loss (Weatherby and Brophy, 2019). Additionally, studies by Dan Lv have shown that anxiety and depression in breast cancer patients during chemotherapy are correlated with the occurrence of adverse drug reactions (Lv et al., 2023). In this study, intervention group patients received more pharmaceutical support and psychological encouragement during follow-up, alleviating their fear and anxiety about adverse drug reactions. This positive mindset facilitated better coping with potential adverse drug reactions such as insomnia and anxiety during treatment, reducing their incidence and severity.

The analysis of medication knowledge scores showed that both groups demonstrated improvements over the 6-month period, while the intervention group exhibited significantly greater gains than the control group, with a statistically significant time × group interaction effect (*P* < 0.001). This finding aligns with previous research (Montagnese et al., 2020; Başoğlu and Polat, 2024). Notably, the control group also experienced score improvements despite lacking additional interventions, which we attribute to the verbal education patients received during clinical visits. As Eric Vachon et al. identified (Vachon et al., 2019), verbal education provided by oncology healthcare providers during each patient encounter can enhance disease-related knowledge scores. Additionally, when control group patients actively queried questionnaire content or related knowledge, pharmacists provided guidance and education for ethical considerations.

In this study, the breast cancer medication knowledge questionnaire was integrated into the software platform for self-administered completion by patients. The user-friendly question-answering format significantly boosted patient participation. Pharmacists reviewed, recorded, and analyzed responses in the backend, identifying gaps in patients’ diagnostic and treatment knowledge to deliver targeted explanations and guidance during follow-up sessions. Interactive quizzing not only effectively assessed patients’ grasp of disease-related knowledge but also subtly fostered their understanding of health status and self-management capabilities. As prior studies have shown, this learning approach significantly enhances patient engagement and has yielded positive outcomes in multiple investigations (Graetz et al., 2024; Jiang et al., 2024; Lally et al., 2024). Furthermore, research indicates that progressive knowledge quizzes can strengthen patients’ confidence in managing their own conditions, playing a vital role in promoting the recovery process (Børøsund et al., 2014).

Overall, the intervention group demonstrated higher medication adherence scores than the control group, with a significant interaction effect between group and time (*P =* 0.021). Further comparative analysis revealed a continuous upward trend in medication adherence scores in the intervention group, indicating that the intervention’s impact gradually emerged and strengthened over time, continuously improving patients’ medication adherence. This is consistent with findings from previous multimodal intervention studies (Xie et al., 2017; Getachew et al., 2022; Li et al., 2024). In contrast, the control group’s medication adherence scores showed a trend of initial increase followed by decrease, which may reflect the influence of multiple factors on patients’ medication adherence without specific interventions, making it difficult to maintain a high level. Such fluctuations are common in routine care without intervention—for example, Sefonias Getachew et al. found that cancer patients’ adherence may improve initially due to high disease awareness but decline over time (Getachew et al., 2022).

In previous studies on chronic diseases, treatment adherence in control groups typically decreases gradually (Bail et al., 2020; Doe et al., 2020; Liu et al., 2020). Notably, both groups in this study showed improved adherence during the first 3 months, which we hypothesize may be due to the following: At baseline, patients were in the early postoperative period, and doctors prescribed painkillers and antibiotics based on their conditions, with nursing staff conducting inquiries and supervision, directly enhancing medication adherence. By the third month, most patients were in the radiotherapy or chemotherapy phase, and due to the periodic nature of breast cancer treatment (Ashok Kumar et al., 2019), patients generally maintained high treatment adherence. By the sixth month, patients had completed most of their treatment and entered the home rehabilitation phase. At this point, without direct medical supervision, the intervention group’s continuous follow-up likely played a critical role in improving medication adherence, whereas the control group’s adherence declined. These results highlight the importance of sustained intervention, particularly in the late treatment phase when patients transition from a hospital to a home environment—continuous pharmaceutical support and follow-up are essential for maintaining medication adherence.

Considering the complexity of oncology treatment, this study adopted the EORTC QLQ-C30 quality of life scale developed by the European Organization for Research and Treatment of Cancer (EORTC) to comprehensively evaluate patients’ quality of life across four dimensions: global health, functional scores, symptom scores, and overall composite scores (Cocks et al., 2023). Overall, the results of each dimension showed distinct characteristics: global health scores initially decreased before increasing over time; functional scores gradually rose; symptom scores increased first and then decreased; and overall composite scores in both groups followed a pattern of initial decline followed by recovery. Notably, the intervention group outperformed the control group in symptom scores and overall composite scores, with quality of life demonstrating different characteristics at each follow-up stage.

Early treatment phase (within 2 months postoperatively): Patients had just completed surgery, and wound pain and drainage tube constraints resulted in lower functional scores, consistent with research on early postoperative functional recovery showing that most breast cancer patients experience varying degrees of limb mobility impairment within 2 months after surgery (Koehler et al., 2015; Alabaster et al., 2021; Akezaki et al., 2023). However, since radiotherapy and chemotherapy had not yet begun, symptom scores were relatively low, and overall quality of life remained high. This suggests that surgery itself had a more pronounced direct impact on functional scores, but other aspects of quality of life were less affected without the interference of chemo/radiotherapy side effects.

Mid-follow-up phase (3–4 months postoperatively): Patients exhibited lower quality of life and more symptoms, primarily due to the peak period of chemotherapy, during which drug-related adverse drug reactions occurred frequently. Multiple studies have shown that quality of life is generally poor during chemotherapy (Pearman, 2003; Fu et al., 2022; Nguyen et al., 2023). At this stage, the adverse reaction of chemotherapy may have partially masked the expected benefits of the intervention, leading to nonsignificant differences in quality of life between the intervention and control groups.

Late follow-up phase (5–6 months postoperatively): Symptom scores significantly decreased in both groups, while global health and functional levels continued to rise, likely due to the completion of chemo/radiotherapy phases and subsequent improvement in quality of life. Additionally, the intervention group’s overall quality of life at 6 months was significantly better than that of the control group, possibly due to the intervention’s positive effects in alleviating symptoms and reducing adverse drug reactions. As a composite endpoint integrating global health, functional scores, and symptom scores, the overall composite scores ultimately also showed intergroup differences, further confirming the effectiveness of the intervention.

This research provides new perspectives and methodologies for the treatment and rehabilitation of non-metastatic breast cancer patients in the field of pharmaceutical care. First, it innovates the research perspective. By focusing on non-metastatic breast cancer patients as the target population for pharmaceutical follow-up services, this study addresses a critical gap in existing literature. Most previous medication-related research has concentrated on inpatients or advanced breast cancer patients, often overlooking the post-discharge medication needs of non-metastatic patients. In reality, this large patient population has long survival periods and substantial medication requirements. By centering on this group, the study fills a void in pharmaceutical care for post-discharge management of non-metastatic breast cancer patients.

Second, it introduces an innovative management model. Traditional follow-up for oncology patients primarily relies on physicians and nurses, frequently lacking pharmacists’ specialized medication guidance—particularly for discharged patients, who face significant unmet needs. This study establishes a new pharmaceutical follow-up service model, leveraging software to enable convenient remote follow-up. Services include adverse reaction monitoring, medication education, prescription simplification, drug interaction screening, and pharmaceutical knowledge dissemination through interactive quizzes, ensuring patients maintain close contact with the care team after discharge. This innovative follow-up model has been shown to improve outcomes and quality of life for non-metastatic breast cancer patients by addressing their unique post-treatment needs.

## 5 Limitations

Although this study demonstrates the positive impact of pharmaceutical follow-up services in reducing the incidence of certain adverse drug reactions, several limitations warrant further exploration and improvement.

First, limitations in preventive measures for hematological adverse drug reactions. Current preventive strategies primarily target non-hematological adverse drug reactions (e.g., ice caps for alopecia, sunscreen for skin reactions, frequent small meals for gastrointestinal symptoms), which effectively prevent or alleviate related symptoms to some extent. However, preventive measures for hematological adverse drug reactions remain limited, largely relying on reactive interventions after mild adverse drug reactions occur (e.g., dose adjustment or growth factor support) to avoid symptom progression. This passive approach cannot achieve pre-emptive intervention, leading to gaps in preventing severe hematological events. The occurrence of hematological adverse drug reactions is closely linked to multiple factors such as individual patient variability and drug-specific properties, with complex mechanisms that increase prevention challenges. Future research should deeply investigate the characteristics and mechanisms of hematological adverse drug reactions and develop more effective preventive strategies—for example, using genetic testing or biomarker monitoring to identify high-risk populations in advance and formulate proactive prevention plans.

Second, limitations in the study duration. The follow-up period in this study was set at 6 months, primarily due to practical considerations regarding sample size and the research team’s time and resource constraints. Although 6 months is a reasonable duration, a small proportion of patients had not completed standard radiotherapy or chemotherapy regimens by 6 months postoperatively, introducing uncertainty about the accuracy of trial results. Additionally, some adverse drug reactions (e.g., ipsilateral limb edema, peripheral neurotoxicity, drug-induced obesity) have longer onset and progression cycles. These symptoms may not have fully manifested or peaked within the 6-month period, potentially masking the intervention’s true effects. To improve the generalizability and accuracy of research findings, future studies should extend the follow-up period to 1 year or longer—where conditions permit—to provide more comprehensive and robust evidence.

## 6 Conclusion

Given the severe incidence of non-metastatic breast cancer and the urgent need for post-discharge follow-up and guidance, exploring remote pharmaceutical follow-up services is of great significance. Focusing on the treatment of non-metastatic breast cancer, this prospective randomized controlled trial found that follow-up services provided by pharmacists significantly reduced the incidence of severe adverse drug reactions and improved patients’ knowledge level, medication adherence, and quality of life. This follow-up model offers a new pathway for the treatment and rehabilitation of non-metastatic breast cancer patients, demonstrating pharmacists’ unique value in the healthcare team and providing insights for optimizing out-of-hospital follow-up care for this patient population.

## Data Availability

The original contributions presented in the study are included in the article/Supplementary Material, further inquiries can be directed to the corresponding author.

## References

[B1] Aapro M. Scotté F. Escobar Y. Celio L. Berman R. Franceschetti A. (2021). Practice patterns for prevention of chemotherapy-induced nausea and vomiting and antiemetic guideline adherence based on real-world prescribing data. Oncologist 26, e1073–e1082. 10.1002/onco.13716 33555084 PMC8176972

[B2] Abousheishaa A. A. Sulaiman A. H. Huri H. Z. Zaini S. Othman N. A. Bin Aladdin Z. (2020). Global scope of hospital pharmacy practice: a scoping review. Healthc. (Basel) 8, 143. 10.3390/healthcare8020143 32466229 PMC7349332

[B3] Akezaki Y. Nakata E. Kikuuchi M. Tominaga R. Kurokawa H. Okamoto M. (2023). Characteristics of postoperative patients with breast cancer aged 65 years and older. Curr. Oncol. 30, 673–680. 10.3390/curroncol30010052 36661701 PMC9858311

[B4] Alabaster K. Homsy C. Puyana S. Higgins M. Ferrin P. Mulcahey M. K. (2021). Exercise after breast reconstruction surgery: evaluating current trends and practices of U.S. plastic surgeons. Plast. Reconstr. Surg. Glob. Open 9, e3857. 10.1097/GOX.0000000000003857 34646725 PMC8500580

[B5] Ashok Kumar P. V. Dakup P. P. Sarkar S. Modasia J. B. Motzner M. S. Gaddameedhi S. (2019). It’s about time: advances in understanding the circadian regulation of DNA damage and repair in carcinogenesis and cancer treatment outcomes. Yale J. Biol. Med. 92, 305–316. 31249491 PMC6585512

[B6] Bail J. R. Ivankova N. Heaton K. Vance D. E. Triebel K. Meneses K. (2020). Cancer-related symptoms and cognitive intervention adherence among breast cancer survivors: a mixed-methods study. Cancer Nurs. 43, 354–365. 10.1097/NCC.0000000000000700 30950929

[B7] Başoğlu S. Polat Ü. (2024). The effect of education and monitoring via tele-nursing to elderly cancer patients using oral anticancer agents on self-efficacy and medication adherence: a randomized controlled trial. Semin. Oncol. Nurs. 40, 151692. 10.1016/j.soncn.2024.151692 39030135

[B8] Børøsund E. Cvancarova M. Moore S. M. Ekstedt M. Ruland C. M. (2014). Comparing effects in regular practice of e-communication and web-based self-management support among breast cancer patients: preliminary results from a randomized controlled trial. J. Med. Internet Res. 16, e295. 10.2196/jmir.3348 25525672 PMC4285721

[B9] Cocks K. Wells J. R. Johnson C. Schmidt H. Koller M. Oerlemans S. (2023). Content validity of the EORTC quality of life questionnaire QLQ-C30 for use in cancer. Eur. J. Cancer 178, 128–138. 10.1016/j.ejca.2022.10.026 36436330

[B10] Doe S. Petersen S. Buekers T. Swain M. (2020). Does a multidisciplinary approach to invasive breast cancer care improve time to treatment and patient compliance? J. Natl. Med. Assoc. 112, 268–274. 10.1016/j.jnma.2020.03.010 32291070 PMC10231650

[B11] Dowling G. P. Daly G. R. Keelan S. Boland F. Toomey S. Hill A. D. K. (2023). Efficacy and safety of trastuzumab deruxtecan in breast cancer: a systematic review and meta-analysis. Clin. Breast Cancer 23, 847–855.e2. 10.1016/j.clbc.2023.09.005 37775347

[B12] Fu L. Feng X. Jin Y. Lu Z. Li R. Xu W. (2022). Symptom clusters and quality of life in gastric cancer patients receiving chemotherapy. J. Pain Symptom Manage 63, 230–243. 10.1016/j.jpainsymman.2021.09.003 34537311

[B13] Getachew S. Addissie A. Seife E. Wakuma T. Unverzagt S. Jemal A. (2022). Breast nurse intervention to improve adherence to endocrine therapy among breast cancer patients in south Ethiopia. Oncologist 27, e650–e660. 10.1093/oncolo/oyac081 35524760 PMC9355816

[B14] Gonçalves J. R. Ramalhinho I. Sleath B. L. Lopes M. J. Cavaco A. M. (2021). Probing pharmacists’ interventions in long-term care: a systematic review. Eur. Geriatr. Med. 12, 673–693. 10.1007/s41999-021-00469-5 33743169

[B15] Graetz I. Hu X. Kocak M. Krukowski R. A. Anderson J. N. Waters T. M. (2024). Remote monitoring app for endocrine therapy adherence among patients with early-stage breast cancer: a randomized clinical trial. JAMA Netw. Open 7, e2417873. 10.1001/jamanetworkopen.2024.17873 38935379 PMC11211959

[B16] Guo Z. Fu L. Chu Z. Gao S. Lu L. Jiao W. (2024). Bibliometric and visual analysis of medication therapy management from 2003 to 2023. Med. (Baltimore) 103, e40953. 10.1097/MD.0000000000040953 39705411 PMC11666177

[B17] Gushrowski M. Rush M. J. Kier K. L. Hinson J. (2025). Description of a pharmacist-led employee wellness hypertension program utilizing remote monitoring devices. Am. J. Health Syst. Pharm., zxaf084. 10.1093/ajhp/zxaf084 40205906

[B18] Hashimoto H. Abe M. Tokuyama O. Mizutani H. Uchitomi Y. Yamaguchi T. (2020). Olanzapine 5 mg plus standard antiemetic therapy for the prevention of chemotherapy-induced nausea and vomiting (J-FORCE): a multicentre, randomised, double-blind, placebo-controlled, phase 3 trial. Lancet Oncol. 21, 242–249. 10.1016/S1470-2045(19)30678-3 31838011

[B19] Hepler C. D. Strand L. M. (1990). Opportunities and responsibilities in pharmaceutical care. Am. J. Hosp. Pharm. 47, 533–543. 10.1093/ajhp/47.3.533 2316538

[B20] Ho P. J. Gernaat S. A. M. Hartman M. Verkooijen H. M. (2018). Health-related quality of life in Asian patients with breast cancer: a systematic review. BMJ Open 8, e020512. 10.1136/bmjopen-2017-020512 29678980 PMC5914715

[B21] Jiang L. Xu J. Wu Y. Liu Y. Wang X. Hu Y. (2024). Effects of the “AI-TA” Mobile app with intelligent design on psychological and related symptoms of young survivors of breast cancer: randomized controlled trial. JMIR Mhealth Uhealth 12, e50783. 10.2196/50783 38833298 PMC11185911

[B22] Kanimozhi R. Padmavathi V. Ramesh P. S. (2025). Perceived digital threats influencing smartphone use among the aging population. Sci. Rep. 15, 27813. 10.1038/s41598-025-12669-1 40738941 PMC12310973

[B23] Kc B. Alrasheedy A. A. Leggat P. A. Mohamed Ibrahim M. I. Christopher C. M. Sapkota B. (2023). Types and outcomes of pharmacist-managed travel health services: a systematic review. Travel Med. Infect. Dis. 51, 102494. 10.1016/j.tmaid.2022.102494 36400319

[B24] Kennedy S. K. F. Goodall S. Lee S. F. DeAngelis C. Jocko A. Charbonneau F. (2024). 2020 ASCO, 2023 NCCN, 2023 MASCC/ESMO, and 2019 CCO: a comparison of antiemetic guidelines for the treatment of chemotherapy-induced nausea and vomiting in cancer patients. Support Care Cancer 32, 280. 10.1007/s00520-024-08462-x 38594320

[B25] Koehler L. A. Blaes A. H. Haddad T. C. Hunter D. W. Hirsch A. T. Ludewig P. M. (2015). Movement, function, pain, and postoperative edema in axillary web syndrome. Phys. Ther. 95, 1345–1353. 10.2522/ptj.20140377 25977305 PMC4595809

[B26] Kwan Y. H. Weng S. D. Loh D. H. F. Phang J. K. Oo L. J. Y. Blalock D. V. (2020). Measurement properties of existing patient-reported outcome measures on medication adherence: systematic review. J. Med. Internet Res. 22, e19179. 10.2196/19179 33034566 PMC7584986

[B27] Lally P. Kennedy F. Smith S. Beeken R. J. Buck C. Thomas C. (2024). The feasibility and acceptability of an app-based intervention with brief behavioural support (APPROACH) to promote brisk walking in people diagnosed with breast, prostate and colorectal cancer in the UK. Cancer Med. 13, e7124. 10.1002/cam4.7124 38529687 PMC10964176

[B28] Lau T. K. H. Yip C. H. W. Yeo W. (2016). State of the art antiemetic therapy for cancer patients. Curr. Oncol. Rep. 18, 2. 10.1007/s11912-015-0486-5 26694923

[B29] Li D. Sun C.-L. Kim H. Soto-Perez-de-Celis E. Chung V. Koczywas M. (2021). Geriatric assessment-driven intervention (GAIN) on chemotherapy-related toxic effects in older adults with cancer: a randomized clinical trial. JAMA Oncol. 7, e214158. 10.1001/jamaoncol.2021.4158 34591080 PMC8485211

[B30] Li H. Sang D. Gong L. Wang B. Wang Y. Jia X. (2024). Improving physical and mental health in women with breast cancer undergoing anthracycline-based chemotherapy through wearable device-based aerobic exercise: a randomized controlled trial. Front. Public Health 12, 1451101. 10.3389/fpubh.2024.1451101 39363984 PMC11446794

[B31] Linsell L. Forbes L. J. L. Burgess C. Kapari M. Thurnham A. Ramirez A. J. (2010). Validation of a measurement tool to assess awareness of breast cancer. Eur. J. Cancer 46, 1374–1381. 10.1016/j.ejca.2010.02.034 20335018

[B32] Liu Y. Zhang Q. Chen Y. Wang C. (2020). Effect of telephone-based health education intervention models on cervical cancer screening compliance: a protocol for systematic review and meta-analysis. Med. (Baltimore) 99, e22130. 10.1097/MD.0000000000022130 33285666 PMC7717719

[B33] Løyland B. Sandbekken I. H. Grov E. K. Utne I. (2024). Causes and risk factors of breast cancer, what do we know for sure? An evidence synthesis of systematic reviews and meta-analyses. Cancers (Basel) 16, 1583. 10.3390/cancers16081583 38672665 PMC11049405

[B34] Lv D. Lan B. Zhang L. Sun X. Yang M. Ma F. (2023). Association between depression and anxiety status of breast cancer patients before adjuvant chemotherapy and chemotherapy-induced adverse events. Cancer Med. 12, 4794–4800. 10.1002/cam4.5283 36161780 PMC9972093

[B35] Ma Z. Shi H. Hong G. Wu L. Lin Y. (2025). Drug-related problems in elderly patients with AECOPD and pharmaceutical intervention practice: a prospective study. Front. Pharmacol. 16, 1596795. 10.3389/fphar.2025.1596795 40612742 PMC12222075

[B36] McKenzie E. Zaki P. Raman S. Olson R. McFarlane T. DeAngelis C. (2019). Radiation-induced nausea and vomiting: a comparison between MASCC/ESMO, ASCO, and NCCN antiemetic guidelines. Support Care Cancer 27, 783–791. 10.1007/s00520-018-4586-2 30607675

[B37] McNab D. Bowie P. Ross A. MacWalter G. Ryan M. Morrison J. (2018). Systematic review and meta-analysis of the effectiveness of pharmacist-led medication reconciliation in the community after hospital discharge. BMJ Qual. Saf. 27, 308–320. 10.1136/bmjqs-2017-007087 29248878 PMC5867444

[B38] Metrebian N. Getty C.-A. Carr E. Weaver T. Pilling S. Kelleher M. (2025). Mobile telephone contingency management to encourage adherence to supervised medication among individuals most at risk of non-adherence to opioid agonist treatment: a study protocol for a feasibility study (TIES2). Pilot Feasibility Stud. 11, 33. 10.1186/s40814-025-01614-8 40128826 PMC11931786

[B39] Montagnese C. Porciello G. Vitale S. Palumbo E. Crispo A. Grimaldi M. (2020). Quality of life in women diagnosed with breast cancer after a 12-Month treatment of lifestyle modifications. Nutrients 13, 136. 10.3390/nu13010136 33396551 PMC7824271

[B40] Morgan E. O’Neill C. Shah R. Langselius O. Su Y. Frick C. (2024). Metastatic recurrence in women diagnosed with non-metastatic breast cancer: a systematic review and meta-analysis. Breast Cancer Res. 26, 171. 10.1186/s13058-024-01881-y 39605105 PMC11603627

[B41] Muntner P. Joyce C. Holt E. He J. Morisky D. Webber L. S. (2011). Defining the minimal detectable change in scores on the eight-item morisky medication adherence scale. Ann. Pharmacother. 45, 569–575. 10.1345/aph.1P677 21521862

[B42] Nguyen L. B. Vu L. G. Le T. T. Nguyen X. T. Dao N. G. Nguyen D. C. (2023). Impact of interventions on the quality of life of cancer patients: a systematic review and meta-analysis of longitudinal research. Health Qual. Life Outcomes 21, 112. 10.1186/s12955-023-02189-9 37821985 PMC10566122

[B43] Papakonstantinou A. Villacampa G. Navarro V. Oliveira M. Valachis A. Pascual T. (2025). Adjuvant endocrine treatment strategies for non-metastatic breast cancer: a network meta-analysis. EClinicalMedicine 81, 103116. 10.1016/j.eclinm.2025.103116 40034565 PMC11875833

[B44] Pearman T. (2003). Quality of life and psychosocial adjustment in gynecologic cancer survivors. Health Qual. Life Outcomes 1, 33. 10.1186/1477-7525-1-33 12952541 PMC194224

[B45] Poggio F. Bruzzone M. Ceppi M. Pondé N. F. La Valle G. Del Mastro L. (2018). Platinum-based neoadjuvant chemotherapy in triple-negative breast cancer: a systematic review and meta-analysis. Ann. Oncol. 29, 1497–1508. 10.1093/annonc/mdy127 29873695

[B46] Roscoe J. A. Heckler C. E. Morrow G. R. Mohile S. G. Dakhil S. R. Wade J. L. (2012). Prevention of delayed nausea: a university of Rochester cancer center community clinical oncology program study of patients receiving chemotherapy. J. Clin. Oncol. 30, 3389–3395. 10.1200/JCO.2011.39.8123 22915657 PMC3438235

[B47] Ruiz-Ramos J. Hernández M. H. Juanes-Borrego A. M. Milà R. Mangues-Bafalluy M. A. Mestres C. (2021). The impact of pharmaceutical care in multidisciplinary teams on health outcomes: systematic review and meta-analysis. J. Am. Med. Dir. Assoc. 22, 2518–2526. 10.1016/j.jamda.2021.05.038 34228962

[B48] Shen J. Ye X. Hou H. Wang Y. (2025). Efficacy and safety of immunochemotherapy in advanced triple-negative breast cancer: a meta-analysis of randomised clinical trials. Clin. Oncol. (R Coll. Radiol) 40, 103783. 10.1016/j.clon.2025.103783 39955967

[B49] Siegel R. L. Miller K. D. Wagle N. S. Jemal A. (2023). Cancer statistics, 2023. CA Cancer J. Clin. 73, 17–48. 10.3322/caac.21763 36633525

[B50] Sweetman S. C. Blake P. S. Brayfield A. McGlashan J. M. (2014). Martindale: the complete drug reference. 37th Edn. Beijing: Chemical Industry Press, 670–671.

[B51] Vachon E. Given B. Given C. Dunn S. (2019). Temporary stoppages and burden of treatment in patients with cancer. Oncol. Nurs. Forum 46, E135–E144. 10.1188/19.ONF.E135-E144 31424460 PMC7360060

[B52] Wang H.-Y. Hang Kwok S. W. Liu X.-L. Wang T. Bressington D. Shen Y. (2023). Quality of life patient/cancer survivor version in Chinese cancer survivors: a validation study. Asia Pac J. Oncol. Nurs. 10, 100255. 10.1016/j.apjon.2023.100255 37519402 PMC10372171

[B53] Wang H. Yao L. Zhong L. Fang J. He Q. Busch T. (2025). Marrow adipogenic lineage precursors (MALPs) facilitate bone marrow recovery after chemotherapy. Bone 195, 117446. 10.1016/j.bone.2025.117446 40057216 PMC12337330

[B54] Weatherby L. Brophy L. (2019). Scalp cooling: a patient’s experience. J. Adv. Pract. Oncol. 10, 158–165. 10.6004/jadpro.2019.10.2.5 31538026 PMC6750918

[B55] Xie F.-L. Wang Y.-Q. Peng L.-F. Lin F.-Y. He Y.-L. Jiang Z.-Q. (2017). Beneficial effect of educational and nutritional intervention on the nutritional status and compliance of gastric cancer patients undergoing chemotherapy: a randomized trial. Nutr. Cancer 69, 762–771. 10.1080/01635581.2017.1321131 28524705

[B56] Yeo W. Lau T. K. Li L. Lai K. T. Pang E. Cheung M. (2020). A randomized study of olanzapine-containing versus standard antiemetic regimens for the prevention of chemotherapy-induced nausea and vomiting in Chinese breast cancer patients. Breast 50, 30–38. 10.1016/j.breast.2020.01.005 31978815 PMC7375549

