# Supplementary figures and images for: Pharmacist-led remote follow-up service for non-metastatic breast cancer patients: a prospective randomised controlled trial of pharmaceutical intervention
Source: Front Pharmacol. 2025 Sep 4;16:1640727. doi: 10.3389/fphar.2025.1640727 (PMC12443711; doi:10.3389/fphar.2025.1640727)

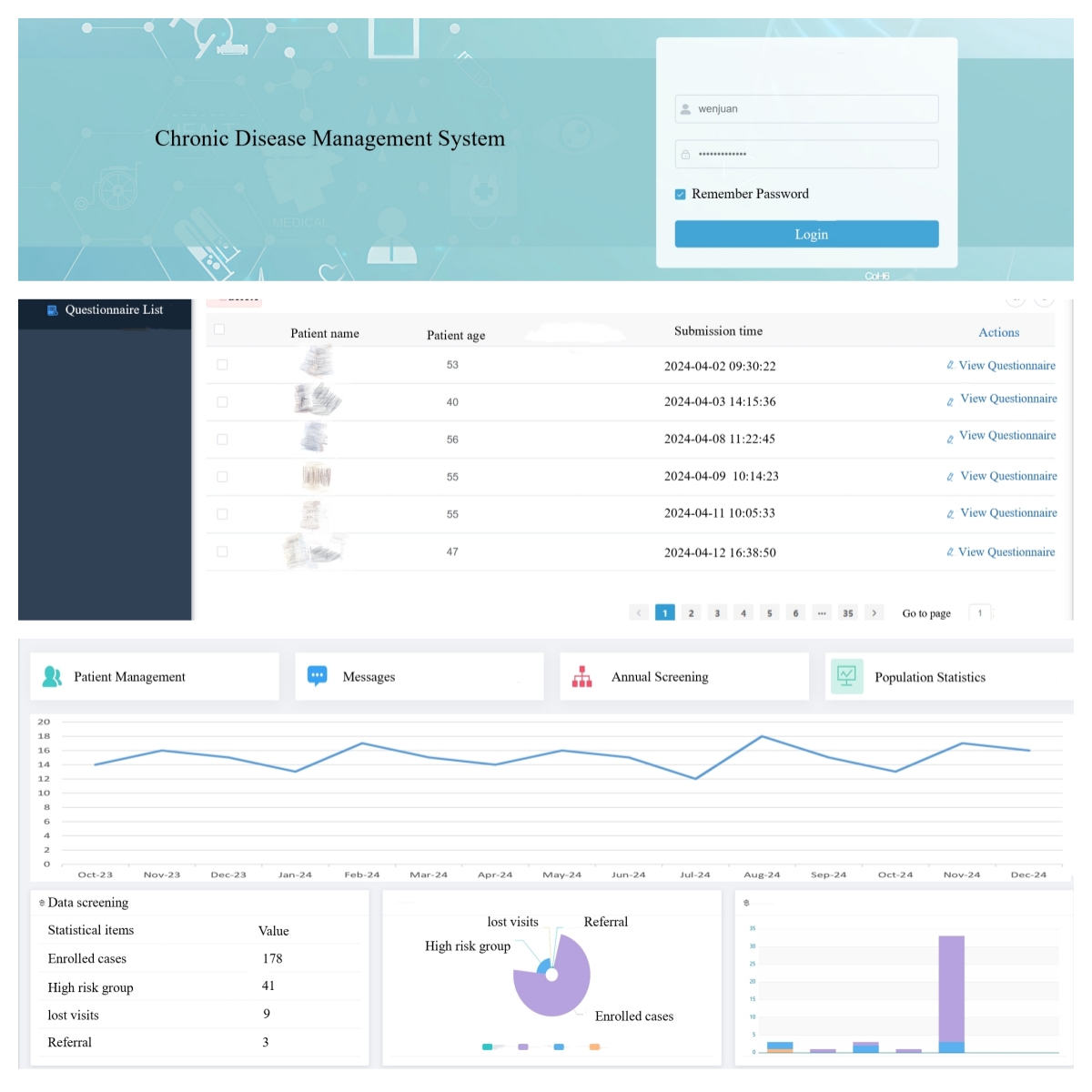

Supplement: Supplementary file 1 [file Supplementaryfile2.jpeg]

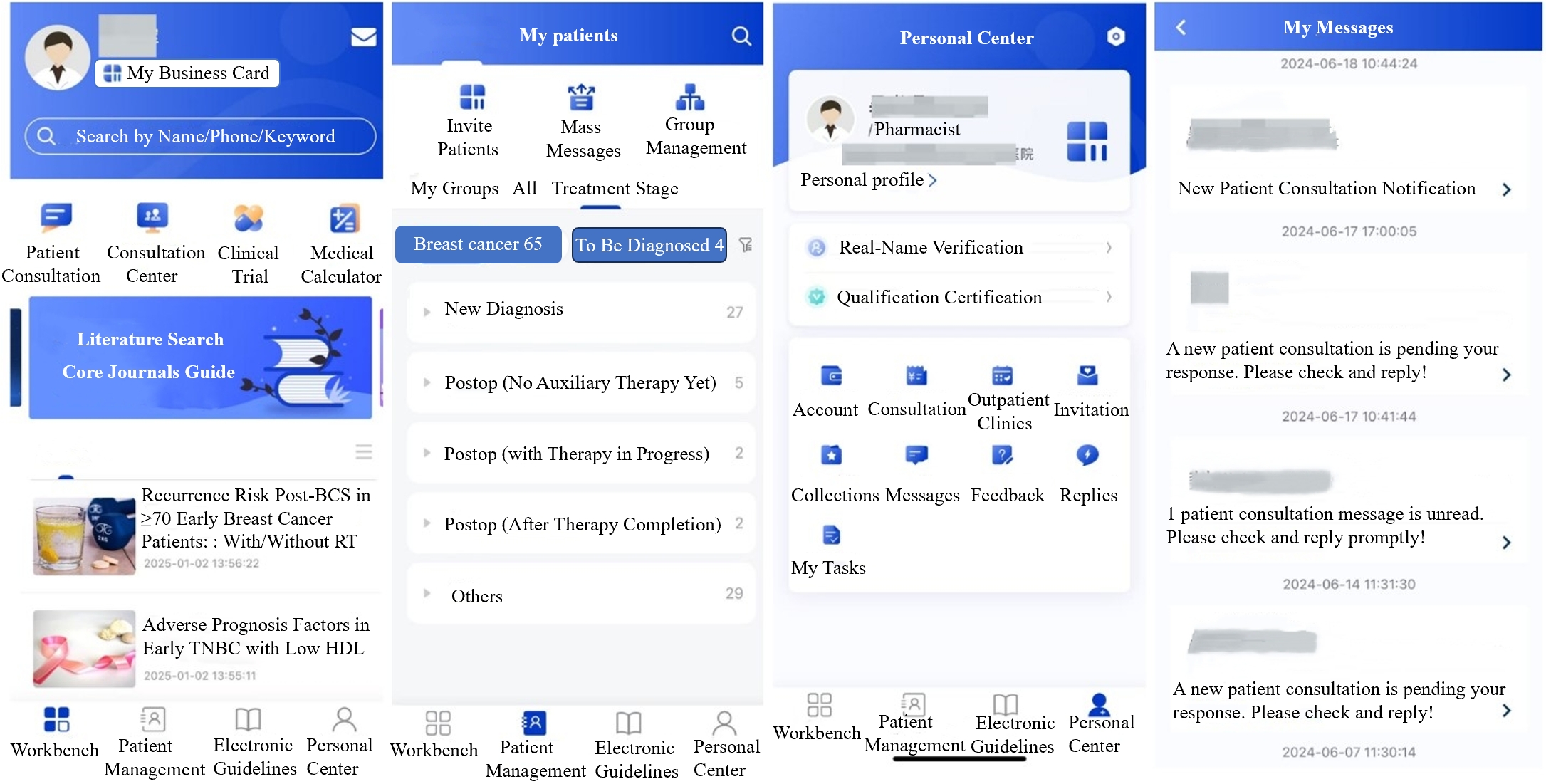

Supplement: Supplementary file 3 [file Supplementaryfile1.jpeg]
