# Supplementary material for: Pharmacist-led remote follow-up service for non-metastatic breast cancer patients: a prospective randomised controlled trial of pharmaceutical intervention
Source: Front Pharmacol. 2025 Sep 4;16:1640727. doi: 10.3389/fphar.2025.1640727 (PMC12443711; doi:10.3389/fphar.2025.1640727)
Supplement: Supplementary file 2 [file Supplementaryfile3.docx]

| **Breast Cancer medication knowledge questionnaire** |  |  |  |
| --- | --- | --- | --- |
| **Single-choice Questions**  **(Please tick “√” in the corresponding box)** | Yes | No | Not Sure |
| 1. Breast cancer patients should avoid using drugs containing estrogen during treatment. | □ | □ | □ |
| 2. Breast cancer incidence risk can be reduced by controlling high-risk factors. | □ | □ | □ |
| 3. Breast cancer patients should avoid taking aspirin during treatment. | □ | □ | □ |
| 4. Age-appropriate childbearing and breastfeeding can reduce the risk of breast cancer. | □ | □ | □ |
| 5. Women aged 35-64 should undergo breast cancer screening at least every 2-3 years. | □ | □ | □ |
| 6. Avoiding obesity and maintaining a healthy weight helps reduce the risk of breast cancer. | □ | □ | □ |
| 7. Breast cancer screening items include breast ultrasound, mammography, breast biopsy, etc. | □ | □ | □ |
| 8. The 5-year survival rate of early breast cancer patients is less than 80%. | □ | □ | □ |
| 9. Breast cancer patients can use traditional Chinese medicine by themselves to enhance immunity during treatment. | □ | □ | □ |
| 10. The use of estrogen-based medications increases the risk of breast cancer recurrence. | □ | □ | □ |
| 11. After surgery, fat-containing foods should be avoided to reduce the risk of recurrence. | □ | □ | □ |
| 12. During treatment, ensure sufficient daily protein intake to support body repair. | □ | □ | □ |
| 13. The affected arm needs sufficient rest, and any activity should be avoided as much as possible. | □ | □ | □ |
| 14. Breast cancer patients should have regular blood tests during chemotherapy. | □ | □ | □ |
| 15. Early postoperative wounds need to be kept dry, and water contact should be avoided as much as possible. | □ | □ | □ |
| 16. Treatment (such as drops and medications) can be taken at convenient times without following the doctor’s advice. | □ | □ | □ |
| 17. Breast hyperplasia, breast cysts, etc., are non-malignant diseases and do not require regular examinations. | □ | □ | □ |
| 18. Maintaining good mental health and reducing stress can lower the probability of disease recurrence. | □ | □ | □ |
| 19. If breast cancer postoperative patients have a fever, they should take antibiotics by themselves. | □ | □ | □ |
| 20. If a rash occurs during oral anti-tumor medication, stop the drug immediately and contact the doctor. | □ | □ | □ |
